# 18F-FDG-PET/CT in relapsed multiple myeloma: Are prognostic thresholds different from first-line therapy?

**DOI:** 10.1186/s12880-022-00788-4

**Published:** 2022-04-04

**Authors:** Romans Zukovs, Christina Antke, Eduards Mamlins, Lino Morris Sawicki, Annemarie Mohring, David Lopez y Niedenhoff, Amelie Boquoi, Mustafa Kondakci, Gerald Antoch, Hans-Wilhelm Müller, Roland Fenk, Rainer Haas

**Affiliations:** 1 Department for Hematology, Oncology and Clinical Immunology, Medical Faculty, Heinrich Heine University Dusseldorf, Moorenstr. 5, 40225 Dusseldorf, Germany; 2 Clinic for Nuclear Medicine, Medical Faculty, Heinrich Heine University Dusseldorf, 40225 Dusseldorf, Germany; 3 Department of Diagnostic and Interventional Radiology, Medical Faculty, Heinrich Heine University Dusseldorf, 40225 Dusseldorf, Germany; 4 Department for Oncology and Hematology, St. Lukas Clinic Solingen, 42697 Solingen, Germany

**Keywords:** Multiple myeloma, Relapse, Prognostic factors, 18FDG PET-CT

## Abstract

**Purpose:**

While ^18^F-FDG PET/CT yields valuable prognostic information for patients in first-line therapy of multiple myeloma (MM), its prognostic relevance in relapse is not established. Available studies of relapsed MM describe prognostic thresholds for frequently used PET/CT parameters that are significantly higher than those identified in the first-line setting. The purpose of this study was to evaluate the prognostic role of PET/CT in relapsed MM, based on parameters used in the first-line setting.

**Methods:**

Our retrospective study included 36 patients with MM who had received autologous or allogeneic stem cell transplantation, suffered at least one relapse, and underwent FDG-PET/CT at relapse. Number of focal bone lesions (FL), maximal standardised uptake value (SUVmax), and presence of PET-positive extramedullary lesions (EMD) were analysed.

**Results:**

For the number of FLs, the prognostic value was demonstrated with a cut-off of > 3 (median OS 3.8 months vs. not reached, p = 0.003). Median OS of patients with SUVmax ≤ 4 was not reached, while it was 3.9 months in patients with SUVmax > 4 (p = 0.014). Presence of EMD was a significant prognostic parameter too, with median OS of 3.6 months versus not reached (p = 0.004). The above-mentioned parameters showed prognostic significance for PFS as well. Combination of higher ISS stage and PET/CT parameters identified patients with particularly short OS (3.7 months vs. not reached, p < 0.001) and PFS (3.6 vs. 11.7 months p < 0.001).

**Conclusion:**

The PET/CT parameters SUVmax > 4, nFL > 3, and presence of EMD identify patients with poor prognosis not only in the first-line setting but also in relapsed MM.

## Background

Multiple myeloma (MM) is a malignant plasma cell disorder that is currently incurable in most cases. The introduction of novel therapeutic agents significantly improved the progression-free and overall survival, but the vast majority of patients will eventually experience disease progression [1, 2]. The multifaceted presentation and progressive nature of MM require complex assessment including detailed radiological examination. Whole-body computed tomography (CT), magnetic resonance imaging (MRI), and positron emission tomography—computed tomography (PET/CT) are the methods of choice, depending on the clinical setting and their availability [3].

The ^18^F-fluorodesoxyglucose (^18^F-FDG) PET/CT is particularly useful for differentiating between active and smoldering myeloma, assessing residual disease after therapy, or detecting extramedullary involvement [4, 5]. Disease activity assessed by PET/CT at diagnosis, after induction therapy, and after high-dose therapy is a useful predictor of progression-free survival (PFS) and overall survival (OS), as shown by multiple prospective and retrospective studies (Table 1). Moreover, comparative pre- and post-therapeutic PET/CT for evaluation of therapy response yields important prognostic information, too [6, 7]. The IMWG consensus statement on radiologic diagnostics in MM recommends the use of PET/CT at diagnosis when available, especially in studies, followed by PET/CT re-evaluation after 1^st^ line treatment for assessment of residual disease [3].

**Table 1:** Overview of studies evaluating prognostic role of variable PET-CT parameters in multiple myeloma

Study	Study design	Number of patients	Time of PET/CT	Thresholds for PET-CT parameters (relevant survival parameter)
Bartel et al. [15]	Prospective	239	At diagnosis and before autologous SCT	nFL > 3 (OS and EFS), SUVmax > 3.9 (EFS*), EMD-positivity (OS* and EFS*)
Zamagni et al. [8]	Prospective	192	At diagnosis	SUVmax > 4.2 (OS and PFS), EMD-positivity (OS and PFS), nFLs > 3 (PFS*)
Fonti et al. [21]	Retrospective	47	At diagnosis, with subpopulation in relapse	MTV (OS and PFS)
Usmani et al. [11]	Prospective	302	At diagnosis and at induction day 7	nFL > 3 on day 7 (OS and PFS)
Zamagni et al. [9]	Retrospective	282	After first line	SUVmax > 4.2 (PFS and OS), nFL > 3 (OS* and PFS*), EMD-positivity (OS* and PFS*)
Patriarca et al. [10]	Retrospective	67	Before allogeneic SCT	SUVmax > 4.2 (OS and PFS*), EMD-positivity (OS* and PFS), nFL > 1 (OS* and PFS*),
Beksac et al. [12]	Prospective	139	Before and after autologous SCT	SUVmax > 3.35 (OS), SUVmax > 4.2 (PFS)
Moreau et al. [22]	Prospective	134	After first line compared to PET at diagnosis	PET-positive versus PET-normalization (PFS and OS)
Davies et al. [14]	Retrospective (based on population of TT4-6 trials)	596	At diagnosis, during and after induction	nFL > 3 (OS, PFS) at diagnosis. Suppression of FL-signal beneficial
Wang et al. [16]	Retrospective	123	At diagnosis	SUVmax > 5.7 (OS*), EMD-positivity (OS and PFS)
Moon et al. [17]	Retrospective	76	At diagnosis	nFL > 3 (OS and PFS), EMD-positivity (OS and PFS)
Fonti et al. [23]	Retrospective	47	At diagnosis	MVT (OS and PFS), TLG (OS* and PFS*), SUVmax (OS*), nFL (OS* and PFS*)
Lapa et al. [18]	Retrospective	37	At relapse	nFL > 10 (TTP and OS), EMD (TTP and OS), SUVmax (ROC) > 18.5 (TTP)
Jamet et al. [19]	Retrospective	40	At relapse	nFL (appendicular skeleton) > 6 (OS, PFS), TLG (OS), SUVmax (ROC) > 15.9 (PFS), nFLs > 13 (PFS*)

*SCT* stem cell transplantation, *OS* overall survival, *EFS* event free survival, *PFS* progression free survival, *CRD* complete response duration, *TTP* time to progression, *nFL* number of focal lesions, *EMD* extramedullary disease, SUV-standardized uptake value, *MTV* metabolic tumor volume, *TLG* total lesion glycolysis. *Parameter was significant only on univariate analysis

Most studies on the prognostic role of PET/CT in MM measured metabolic disease activity using standardized uptake values (SUV) and evaluated the number of FDG-avid focal intramedullary lesions (FL) as well as the presence of extramedullary disease (EMD) [4, 8–16]. Prospective studies during first line therapy identified cut-off values for PET/CT parameters with minor variability [8, 11, 12, 15]. These prognostically significant thresholds were reproduced in multiple smaller retrospective studies (Table 1). A maximal SUV (SUVmax) above 3.9–4.2, more than 3 FLs, and the presence of PET-positive EMD were repeatedly demonstrated to be negative predictors for OS and/or PFS [8–10, 14, 15, 17].

The role of PET/CT in relapsed or refractory MM is less clear, particularly in the post-transplantation setting [4]. The parameters and thresholds used in relapse studies are heterogeneous, which hampers the evaluation of the prognostic role of PET/CT. The prognostic thresholds proposed for SUVmax and number of focal lesions in patients with relapsed MM are 3–4 times higher and more variable than those identified in the first-line setting [18, 19] (Table 1).

In our retrospective single center study, we analyzed patients with MM who underwent at least one HDT with autologous or allogeneic SCT and experienced disease relapse. We evaluated the prognostic role of ^18^F-FDG-PET-CT for OS and PFS, focusing on prognostic parameters that were mostly identified in larger prospective studies looking at baseline parameters and/or response to therapy.

## Methods

We retrospectively evaluated patients with MM who underwent ^18^F-FDG-PET/CT in our center between 2012 and 2019. In this population, 36 pre-treated patients, who had received at least one autologous or allogeneic SCT and experienced disease relapse at the time of PET/CT investigation, were identified and included in the study. Relapse was defined according to IMWG-criteria [20]. Patients with relapse who had already started new therapy prior to the PET/CT were excluded. Clinical findings and laboratory results were integrated into our analysis (Table 2).

**Table 2:** Overview of patient’s data

Parameter	Number (%)
Patients, total	36
Age, years, median	60
Age, years, range	44–76
Sex, male	20 (55.5%)
Sex, female	16 (44.5%)
MM subtype	
IgG Kappa/Lambda	12 (33.3%)/7 (19.4%)
IgA Kappa/Lambda	3 (8.3%)/2 (5.6%)
IgM Lambda	1 (2.8%)
Light chain only, Kappa	5 (13.9%)
Light chain only, Lambda	3 (8.3%)
Non-secretory	3 (8.3%)
High-dose therapies with SCTs	
One autologous SCT	17 (47.2%)
Two autologous SCT	12 (33.3%)
Three autologous SCT	1 (2.8%)
Allogeneic SCT (following autologous)	6 (16.7%) [5(13.9%)]
Therapy lines before current progress	
First line	13 (36.1%)
Second line	9 (25%)
Third line	8 (22.2%)
Fourth line	4 (11.1%)
Fifth line	2 (5.6%)
Median	2
ISS^2^ stage at current progress	
I	19 (52.8%)
II	13 (36.1%)
III	4 (11.1%)
Cytogenetics on FISH	
High-eisk	6 (16.6%)
Non-high-risk	15 (41.7%)
Cytogenetics not available	15 (41.7%)
LDH	
Elevated (> 250 U/l)	20 (55.5%)
Normal (≤ 250 U/l)	16 (44.5%)
Best response after last therapy line	
CR	10 (27.7%)
vgPR	5 (13.9%)
PR	19 (52.8%)
SD	2 (5.6%)

*SCT* stem cell transplantation, *ISS* international staging system, *FISH* fluorescence in situ hybridization, *CR* complete remission, *vgPR* very good partial remission, *PR* partial remission, *SD* stable disease

### PET-CT Scans

All scans were performed as a non-contrast low-dose PET/CT (mCT, 128 slices, Siemens Healthineers). Scan range included whole body from the top of the head through the feet. The ^18^F-FDG-tracer dosing was adapted for body weight (3MBq ^18^F-FDG/kg). The visual evaluation of the PET was carried out using the Ultra-HD-3D mode reconstruction and the iterative 3D-mode reconstructions. The SUV measurements were conducted using the iterative 3D-mode reconstructions. The SUVmax was defined as maximal measured SUV value of the single most active intramedullary or extramedullary lesion on each scan using manually placed volumes of interest (VOI). Focal bone lesions were defined as FDG-avid focal areas within bone structure with tracer uptake intensity above median bone marrow activity (based on the prospective study by Bartel et al. [15]). The threshold of more than 3 focal lesions and the SUVmax cut-off value of 4 for survival analysis were based on prospective and larger retrospective studies of ^18^F-FDG-PET/CT prognostic role in first-line therapy (Table 1) and IMWG 2017 consensus statement [4]. The presence of PET-positive EMD was also evaluated. EMD was defined as FDG-avid lesion that was not contiguous to bone on CT-scan and arose in soft tissue.

### Statistics

We used commercial software (SPSS ver. 23 by IBM Statistics) for statistical analysis. Kaplan–Meier survival analysis was performed to assess the time of OS and PFS with start-point at the moment of the PET/CT investigation. Survivors were censored at the time of the last clinical contact. In survival analysis, an event was defined as death of any reason for OS, and as next disease relapse or death for PFS. Log-rank tests were used to assess statistical significance. A significance level of p-value was set at 0.05. Besides PET parameters, clinical findings and laboratory results, as well as number of prior treatment lines, were evaluated as risk factors.

## Results

### Patient characteristics

Among 36 relapsed patients, 20 were male and 16 female (Table 2). Median age was 60 years (range 44–76). Seventeen patients previously underwent single autologous SCT, 12 patients received two autologous SCTs, and 1 patient had three autologous SCTs. Six patients underwent allogeneic SCT, five of them after previous autologous transplantation. Thirteen (36%) patients experienced first disease relapse, 9 (25%) had a second, 8 (22%) had a third, and 6 (17%) patients suffered a fourth or fifth relapse. With a median follow-up of 37.9 months, the median OS in the entire study population was 16.3 (SE 17.7) months. Median time of PFS of was 6.0 (SE 3.6) months. Five of 36 patients (14%) were lost to follow-up before reaching next progress or death.

### PET parameters

At the time of relapse, 26/36 (72.2%) of our patients had a positive PET scan. Among those, 14/26 patients had only intramedullary disease, while 10/26 had both intra- and extramedullary FDG-avid lesions. Two patients had EMD only. Ten patients had a negative PET scan with no FDG-avid lesions detectable.

### Maximal SUV

**Fig. 1:**
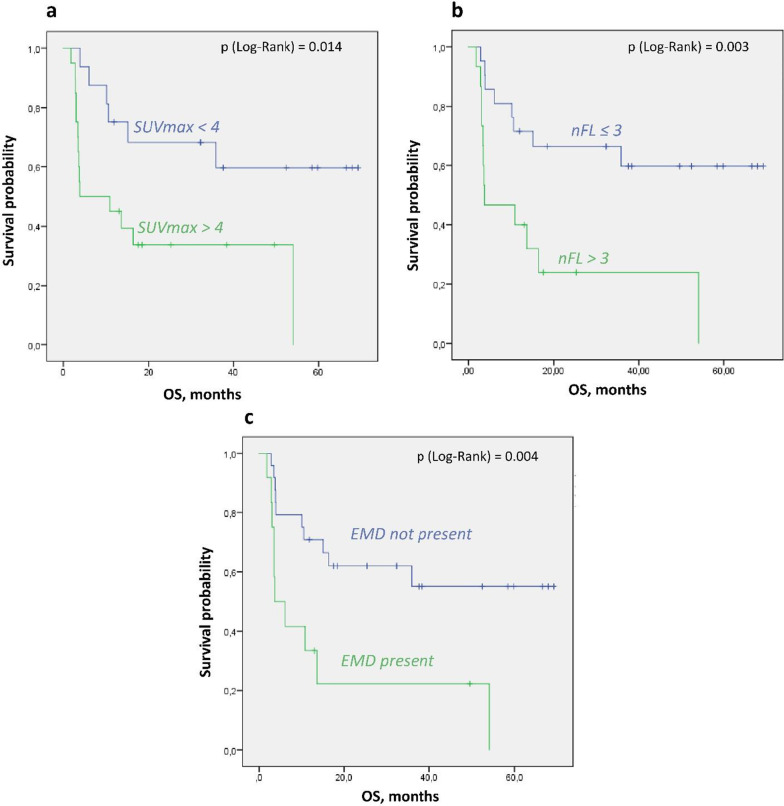
Kaplan–Meier analysis of prognostic impact of PET-CT parameters for overall survival. **a** According to maximum SUV over 4. **b** According to number of focal lesions, threshold over 3. **c** According to presence of EMD

**Fig. 2:**
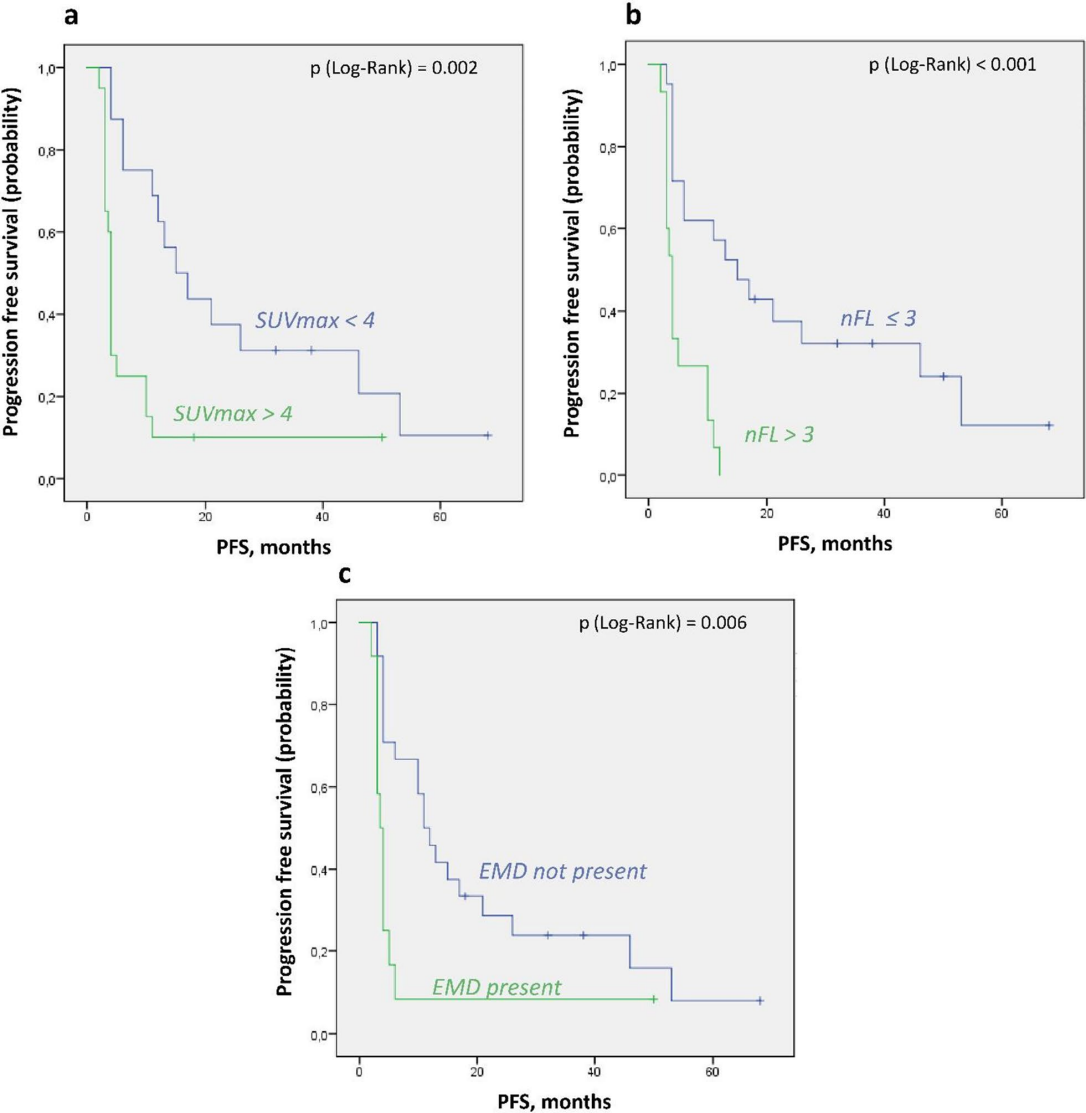
Kaplan–Meier analysis of prognostic impact of PET-CT parameters for progression free survival. **a** According to maximum SUV over 4. **b** According to number of focal lesions, threshold over 3. **c** According to presence of EMD

The median OS of the 16 patients with an SUVmax < 4 was not reached, while in the 20 patients with an SUVmax > 4 the estimated median survival was 3.9 (SE 8.01) months (p = 0.014, Fig. 1a). Using established first-line parameters, a significantly shorter time of PFS was observed in 20 patients with SUVmax > 4 (median of 4 (SE 0.17) vs. 15 (SE 4) months, p = 0.002) as well (Fig. 2a).

### Focal bone lesions

The presence of a single PET-positive focal lesion failed to achieve significance for OS in our population (p = 0.1). Looking at the established number of FL in first-line patients, the median survival of the 15 patients with more than 3 FLs was significantly shorter (3.8 months, SE 4.7) than in 21 patients with 3 or less FLs (median not reached, p = 0.003, Fig. 1b).

Similarly, the presence of a single PET-positive focal lesion was not a significant predictor for shorter PFS (p = 0.08), while the threshold of > 3 focal lesions achieved significance as negative predictor for PFS (3.5 (SE 0.14) vs. 15 (SE 3.8) months, p < 0.001) (Fig. 2b). Interestingly, the presence of > 3 focal bone lesions on CT alone, without PET assessment of lesion activity, had no statistical significance as a negative predictor of OS or PFS (Table 3).

**Table 3:** The results of statistical analysis

Parameter	Patients, n	For OS		For PFS	
		p-value, log-rank	Hazard ratio (CI 95%)	p-value, log-rank	Hazard ratio (CI 95%)
*PET/CT-parameters*					
SUVmax > 4	20/36	**0.014**	3.19 (1.2–8.48)	**0.002**	3.01 (1.39–6.51)
n, FLs ≥ 1	23/36	0.10	2.29 (0.83–6.32)	0.08	1.86 (0.88–3.93)
n, FLs > 3	15/36	**0.003**	3.7 (1.48–9.27)	** < 0.001**	5.54 (2.25–13.6)
EMD Present versus not present	12/36	**0.004**	3.40 (1.39–8.30)	**0.006**	2.67 (1.23–5.83)
n, FLs > 3 on CT only*	28/36	0.74	1.19 (0.39–3.59)	0.54	1.31 (0.53–3.22)
*Remission status*					
PR or worse versus at least vgPR	21/36	0.327	1.58 (0.63–3.97)	0.376	1.38 (0.67–2.83)
*Prior therapy lines*					
≥ 2	23/36	0.265	1.76 (0.64–4.87)	0.128	1.84 (0.83–4.07)
≥ 3	14/36	0.72	2.2 (0.91–5.4)	**0.001**	3.6 (1.59–8.13)
*Clinical parameters*					
Age (≥ 65 years)	11/36	0.169	1.86 (0.75–4.58)	0.082	1.87 (0.86–4.07)
Elevated LDH (> 250 U/l)	20/36	**0.006**	3.86 (1.38–10.77)	**0.003**	2.89 (1.35–6.17)
ISS-Stage (I vs. II vs. III)	19/13/4	**0.016**	2.37 (1.26–4.44)	**0.046**	1.88 (1.09–3.24)

The statistically significant results with p-value below 0.05 are highlighted in bold

*OS* overall survival, *PFS* progression free survival, *SUV* standardized uptake value, *FLs* focal (bone) lesions, *EMD* extramedullary disease, *PR* partial remission, *vgPR* very good partial remission, *FLC* free light chains. *Over 3 bone lesions on CT without PET-positivity

### Extramedullary disease

FDG-avid EMD was present in 12/36 (33.3%) of our patients. In patients with EMD the median OS was 3.63 (SE 2.2) months, compared to not reached in patients with no EMD on PET/CT (p = 0.004, Fig. 1c). In patients with EMD the median PFS was 3.5 (SE 0.4) months, compared to 11 (SE 1.8) months in patients with no EMD on PET-CT (p = 0.006, Fig. 2c). In 9/12 cases the SUVmax was located in the extramedullary lesion and in 8 of those 9 cases it exceeded the threshold of 4. In 8 of 12 patients radiation therapy of PET-positive extramedullary lesions was initiated, demonstrating relevance for therapy planning.

### Laboratory and clinical parameters

**Fig. 3:**
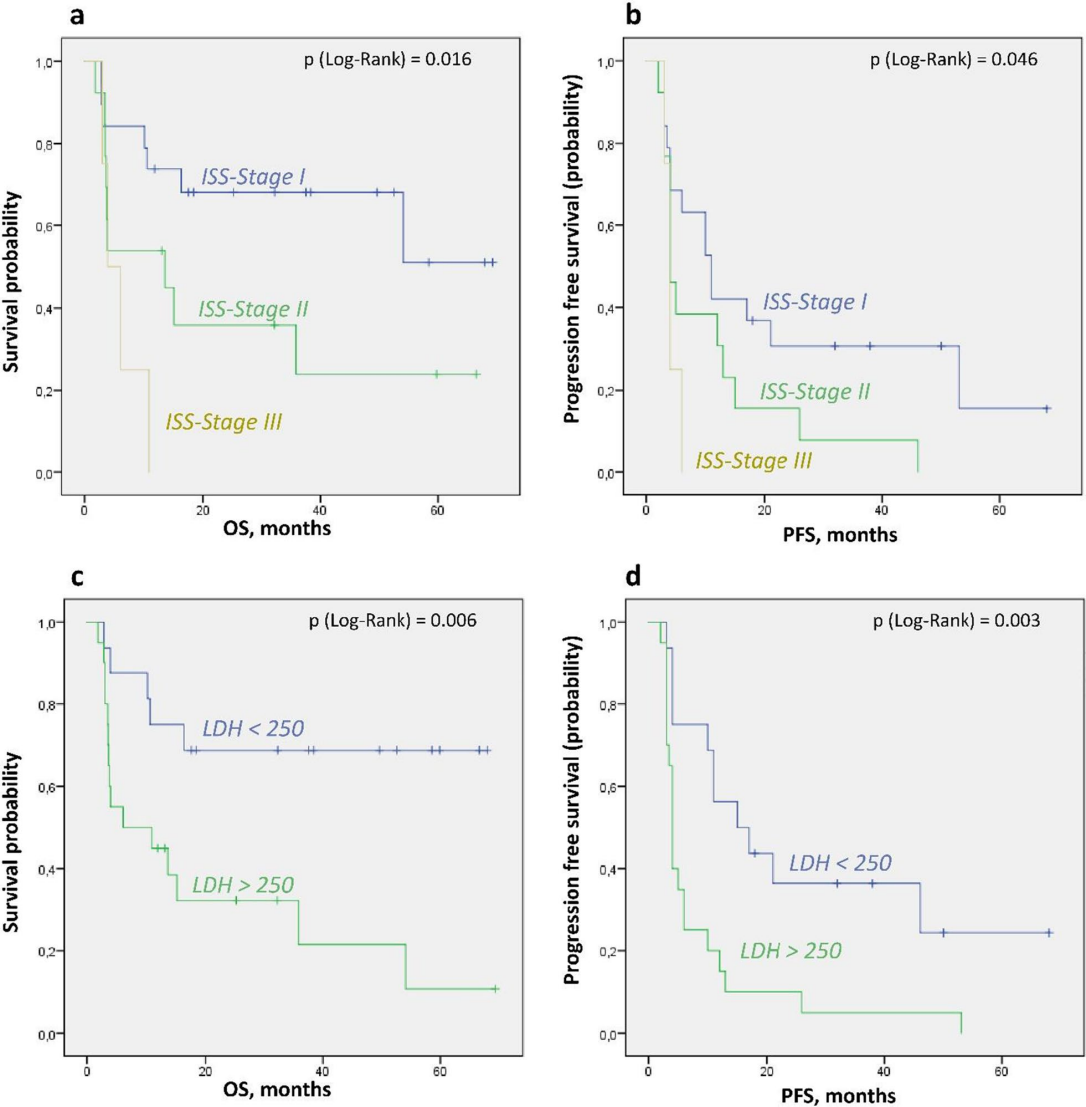
Kaplan–Meier analysis of prognostic impact of serum LDH levels and ISS-Stage for OS and PFS. **a** According to ISS—Stage (OS), **b** according to ISS—Stage (PFS), **c** according to LDH, threshold > 250 U/l (OS), **d** according to LDH, threshold > 250 U/l (PFS)

Laboratory parameters were assessed at the time of PET/CT investigation (Table 3). Thirteen patients had ISS stage II and four had stage III. The OS was significantly shorter in patients with higher ISS stage (3.9 and 13.6 months vs. not reached, p = 0.016, Fig. 3a). The ISS stage had somewhat weaker, but still significant negative predictive value for PFS as well (p = 0.046, Fig. 3b). In 15/36 patients cytogenetic analysis was not available, precluding determination of disease stage according to the revised ISS (R-ISS). The role of elevated LDH (> 250 U/l), as an element of R-ISS, was assessed separately. LDH was elevated in 20/36 patients and was predictive of significantly shorter OS (6 months vs. not reached, p = 0.006, Fig. 3c) and PFS (4 vs. 15 months, p = 0.003, Fig. 3d). Patients relapsing after third-line or later line of therapy had significantly shorter PFS (p = 0.001) and showed a strong trend to lower OS (p = 0.069).

### Combining PET and ISS

**Fig. 4:**
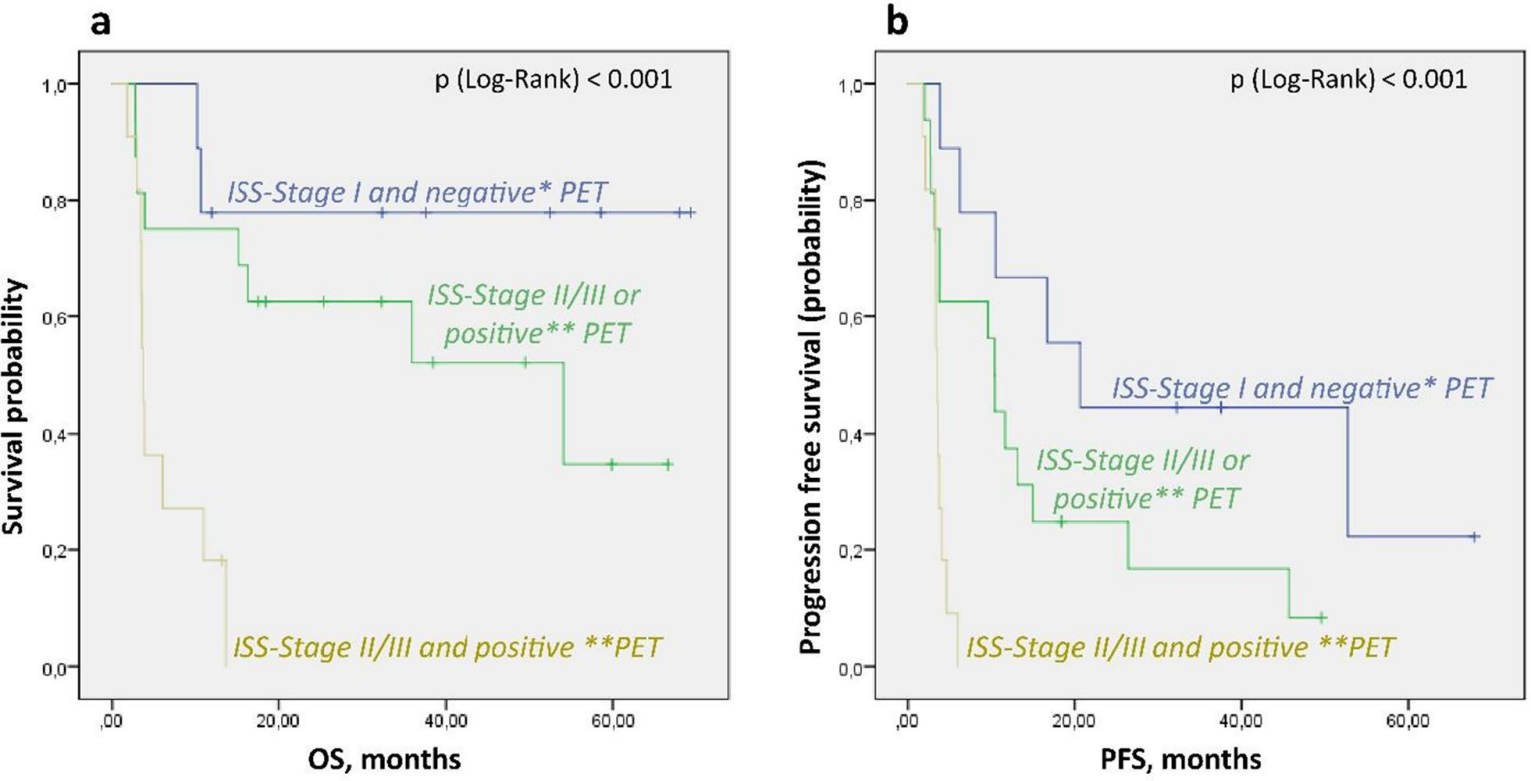
Kaplan–Meier analysis of combined prognostic impact of ISS and PET-Parameters. **a** according to OS, **b** according to PFS. *negative PET—none of established predictive PET-parameters (SUVmax > 4, nFLs > 3, EMD) present. **positive PET—at least one of predictive PET-parameters (SUVmax > 4, nFLs > 3, EMD) present

To identify the patients with the highest risk, we evaluated the combined value of PET and ISS. We selected the cases in which both higher ISS stage (II or III) and at least one of the predictive PET parameters (EMD, nFL > 3, or SUVmax > 4) was present. These “high-risk” patients (n = 11) had significantly shorter OS than the rest of the population (3.7 (SE 0.2) months vs. not reached, p < 0.001), with minimal variability. The PFS was significantly shorter in these patients, too (3.63 (SE 0.14) vs. 11.7 (SE 2.2), p < 0.001). In contrast, the “low-risk” patients with ISS stage I and none of the predictive PET parameters survived significantly longer (OS: not reached vs. 13.6 months, p = 0.028; PFS: 20.7 vs. 5.9 months, p = 0.007). The comparison of “high-risk” versus “low-risk” patients versus others in a single model is demonstrated in Fig. 4.

## Discussion

Evaluation of the prognostic role of PET/CT in the first line setting of myeloma treatment identified useful thresholds for certain parameters. For instance, Bartel et al. [15] showed in a large prospective study of 239 patients that the presence of more than 3 FDG-avid focal bone lesions at baseline was an independent prognostic factor for OS and event-free survival (EFS), while the presence of FDG-avid EMD and a SUVmax > 3.9 were significant on monovariate analysis. These findings were confirmed in a prospective study of 192 patients by Zamagni et al. in 2011. Presence of FDG-avid EMD and SUVmax > 4.2 at baseline were identified as independent negative prognostic factors for OS and PFS [8]. The presence of more than 3 PET-positive FLs at day 7 of induction therapy was an independent negative prognostic parameter for OS and PFS in the large prospective study of 302 patients by Usmani et al. [11]. In 2015, Zamagni et al. demonstrated the negative prognostic role of baseline SUVmax > 4.2 for PFS and OS in a retrospective evaluation of 282 patients [9]. Patriarca et al. [10] demonstrated the negative prognostic role of SUVmax > 4.2 in 67 patients prior to autologous SCT. Davies et al. [14] corroborated the presence of more than 3 PET-positive FLs at baseline as a negative prognostic parameter for OS and PFS in a retrospective evaluation of 596 cases. Wang et al. [16] demonstrated the negative prognostic role of EMD present at baseline for both OS and PFS. The IMWG 2017 consensus statement proposed a threshold of SUV > 4 for defining significant PET-positivity [4].

In comparison, retrospective analyses in patients with relapse after SCT identified significantly higher prognostic thresholds. A study of 40 patients by Jamet et al. selected relapsed patients after high-dose chemotherapy and SCT. A SUVmax > 15.9 and more than 13 FLs (or > 6 FLs in appendicular skeleton) were identified as negative prognostic predictors for PFS [19]. Earlier, Lapa et al. [18] had performed a retrospective analysis of 37 patients suffering relapse after at least one autologous and/or allogeneic SCT. A SUVmax > 18.57 was predictive of shorter time to progression (TTP), while more than 10 FLs and the presence of EMD predicted both shorter TTP and OS. In both studies, patients were heavily pre-treated prior to relapse, which is true for our study as well.

We used a lower threshold of SUVmax > 4, based on the results of larger first line studies. In our relapse population, the SUVmax > 4 had a negative prognostic impact on both OS and PFS. A similar trend was seen regarding cut-offs for active FLs. Multiple pre-SCT studies of patients in first-line therapy identified a threshold of more than 3 FLs as a negative predictor for OS and/or PFS [8, 9, 14, 15, 17]. Similar to SUVmax, the threshold number of FLs with negative prognostic power was much higher (6 or 10 FLs) in retrospective studies of relapsed MM [18, 19]. However, the results of our analysis support the prognostic significance of the lower cut-off value of FL > 3 for OS and PFS in patients with relapsed MM. Furthermore, the presence of PET-positive EMD was a significant negative predictor of OS and PFS, in accordance with the results of multiple first line studies, and as demonstrated by Lapa et al. [18] for relapsed myeloma.

While the comparison with previous relapse studies is hampered by small study populations and variability of pretreatment intensity, our findings still provide a basis for comparing prognostic thresholds of PET/CT parameters in the first-line and relapse settings.

ISS stage is the most widely used tool for prognostic assessment and demonstrated prognostic significance for OS and PFS in our patient population. Combining ISS and PET-parameters allowed us to reliably identify patients with particularly short survival. We demonstrated that PET is an independent prognostic parameter, adding prognostic information to ISS stage. The combined use of PET thresholds and clinical parameters may enhance PET-based prognostic evaluation in the first-line and relapse setting.

Our analysis has certain limitations. Similar to other studies of PET/CT in MM relapse after SCT, patients in our study were heterogeneous regarding their pretreatment history. 23 of 36 patients (63.8%) had received at least 2 lines of therapy before current relapse, and 18 of 36 (50%) had received more than one SCT. Since patient selection for our retrospective analysis covered a 7-year period (2012–2019), treatment protocols varied substantially. In addition, small patient numbers render multivariate models less informative and put more emphasis on univariate analysis. Still, the established clinical prognostic parameters, including elevated LDH and higher ISS proved themselves as significant negative predictors for both OS and PFS in our relatively small study population. The applicability of established clinical prognostic parameters suggests that our patient cohort is representative of a wider population of patients with MM and supports the conclusions drawn from this study population.

Our results could contribute to the management of MM at relapse after SCT. Data up to now only showed that very high thresholds for PET-parameters, such as SUVmax of over 15 or 18 and nFLs of over 6 or 10 are prognostically relevant in relapse setting [18, 19]. We show that lower thresholds of FLs > 3 or SUVmax > 4 already identify patients with high risk and therefore should lead physicists to treat these patients more aggressively. The presence of extramedullary disease was a significant negative prognostic parameter both in our population and in previous studies.

## Conclusions

We show that the same PET/CT parameter thresholds that are used in the first-line setting can also stratify patients with relapsed MM and identify those with particularly poor prognosis. Our findings demonstrate the prognostic yield of PET/CT in MM relapse independent of ISS stage and suggest that the same thresholds for PET prognostic parameters can be used at baseline and at later stages in the course of disease.Patients with relapsed MM who show unfavourable prognostic PET/CT parameters may survive only few months. The use of ^18^F-FDG-PET/CT, especially in combination with established clinical parameters, may thus help to select high-risk patients with MM relapse who may benefit from aggressive treatment approaches.

## Data Availability

The anonymised dataset generated and analysed during the current study is available from the corresponding author on reasonable request.

## Abbreviations

^18^F-FDG: Fluorodeoxyglucose F18
CRD: Complete response duration
CR: Complete remission
EFS: Event-free survival
EMD: Extramedullary disease, Extramedullary Lesions
FISH: Fluorescence in situ hybridization
FL: Focal (bone) Lesion
nFLs: Number of focal (bone) Lesions
HDT: High-dose chemotherapy
IMWG: International Myeloma Working Group
ISS: (Multiple myeloma) International Staging System
LDH: Lactate dehydrogenase
MM: Multiple myeloma
OS: Overall survival
PET/CT: Positron emission tomography computed tomography
PFS: Progression-free survival
PR: Partial remission
vgPR: Very good Partial Remission
R-ISS: Revised (multiple myeloma) International Staging System
SCT: Stem cell transplantation
SE: Standard error
SD: Stable disease
SUV: Standardised uptake value
SUVmax: Maximal standardised uptake value
TTP: Time to progression
VOI: Volume of interest

## References

[1] Kumar S, Paiva B, Anderson KC (2016). International Myeloma Working Group consensus criteria for response and minimal residual disease assessment in multiple myeloma. Lancet Oncol.

[2] Röllig C, Knop S, Bornhäuser M (2015). Multiple myeloma. Lancet.

[3] Hillengass J, Usmani S, Rajkumar SV (2019). International myeloma working group consensus recommendations on imaging in monoclonal plasma cell disorders. Lancet Oncol.

[4] Cavo M, Terpos E, Nanni C (2017). Role of 18F-FDG PET/CT in the diagnosis and management of multiple myeloma and other plasma cell disorders: a consensus statement by the International Myeloma Working Group. Lancet Oncol.

[5] Barwick T, Bretsztajn L, Wallitt K, Amiras D, Rockall A, Messiou C (2019). Imaging in myeloma with focus on advanced imaging techniques. Br J Radiol.

[6] Bailly C, Leforestier R, Jamet B (2017). PET imaging for initial staging and therapy assessment in multiple myeloma patients. Int J Mol Sci.

[7] Nanni C, Zamagni E (2017). Therapy assessment in multiple myeloma with PET. Eur J Nucl Med Mol Imaging.

[8] Zamagni E, Patriarca F, Nanni C (2011). Prognostic relevance of 18-F FDG PET/CT in newly diagnosed multiple myeloma patients treated with up-front autologous transplantation. Blood.

[9] Zamagni E, Nanni C, Mancuso K (2015). PET/CT improves the definition of complete response and allows to detect otherwise unidentifiable skeletal progression in multiple myeloma. Clin Cancer Res.

[10] Patriarca F, Carobolante F, Zamagni E (2015). The role of positron emission tomography with 18F-fluorodeoxyglucose integrated with computed tomography in the evaluation of patients with multiple myeloma undergoing allogeneic stem cell transplantation. Biol Blood Marrow Transplant.

[11] Usmani SZ, Mitchell A, Waheed S (2013). Prognostic implications of serial 18-fluoro-deoxyglucose emission tomography in multiple myeloma treated with total therapy 3. Blood.

[12] Beksac M, Gunduz M, Ozen M, Bakanay Ozturk S, Kucuk O, Ozkan E (2014). Impact of PET-CT response on survival parameters following autologous stem cell transplantation among patients with multiple myeloma: comparison of two cut-off values. Blood.

[13] Jung SH, Kwon SY, Min JJ (2019). 18F-FDG PET/CT is useful for determining survival outcomes of patients with multiple myeloma classified as stage II and III with the Revised International Staging System. Eur J Nucl Med Mol Imaging.

[14] Davies FE, Rosenthal A, Rasche L (2018). Treatment to suppression of focal lesions on positron emission tomography-computed tomography is a therapeutic goal in newly diagnosed multiple myeloma. Haematologica.

[15] Bartel TB, Haessler J, Brown TLY (2009). F18-fluorodeoxyglucose positron emission tomography in the context of other imaging techniques and prognostic factors in multiple myeloma. Blood.

[16] Wang C, Wu L, Sun C, Zhang Y (2019). Clinical relevance of PET/CT in patients with newly diagnosed multiple myeloma. Int J Clin Exp Pathol.

[17] Moon SH, Choi WH, Yoo IR (2018). Prognostic value of baseline18F-fluorodeoxyglucose PET/CT in patients with multiple myeloma: a multicenter cohort study. Korean J Radiol.

[18] Lapa C, Lückerath K, Malzahn U (2014). 18FDG-PET/CT for prognostic stratification of patients with multiple myeloma relapse after stem cell transplantation. Oncotarget.

[19] Jamet B, Bailly C, Carlier T (2019). Added prognostic value of FDG-PET/CT in relapsing multiple myeloma patients. Leuk Lymphoma.

[20] Rajkumar SV, Dimopoulos MA, Palumbo A (2014). International Myeloma Working Group updated criteria for the diagnosis of multiple myeloma. Lancet Oncol.

[21] Fonti R, Larobina M, Del Vecchio S (2012). Metabolic tumor volume assessed by18F-FDG PET/CT for the prediction of outcome in patients with multiple myeloma. J Nucl Med.

[22] Moreau P, Attal M, Caillot D (2017). Prospective evaluation of magnetic resonance imaging and [18F]fluorodeoxyglucose positron emission tomography-computed tomography at diagnosis and before maintenance therapy in symptomatic patients with multiple myeloma included in the IFM/DFCI 2009 trial. J Clin Oncol.

[23] Fonti R, Pellegrino S, Catalano L, Pane F, Del Vecchio S, Pace L (2020). Visual and volumetric parameters by 18F-FDG-PET/CT: a head to head comparison for the prediction of outcome in patients with multiple myeloma. Ann Hematol.

